# Focal pulsed field ablation and ultrahigh-density mapping — versatile tools for all atrial arrhythmias? Initial procedural experiences

**DOI:** 10.1007/s10840-023-01570-4

**Published:** 2023-05-30

**Authors:** Martin H. Ruwald, Arne Johannessen, Morten Lock Hansen, Martin Haugdal, Rene Worck, Jim Hansen

**Affiliations:** Division of Electrophysiology, Department of Cardiology, Herlev-Gentofte Hospital, Gentofte Hospitalsvej 1, DK-2900 Hellerup, Denmark

**Keywords:** Pulsed field ablation, Electrophysiology, Ultrahigh density, Mapping, Atrial fibrillation, Safety

## Abstract

**Background:**

Focal pulsed field ablation (FPFA) is a novel and promising method of cardiac ablation. The aim of this study was to report the feasibility, short-term safety, and procedural findings for a broad spectrum of ablated atrial arrhythmias.

**Methods:**

Patients (*n* = 51) scheduled for ablation of atrial arrhythmias were prospectively included and underwent FPFA using the Galvanize CENTAURI generator with energy delivery through commercially available ablation catheters with ultrahigh-density (UHDx) 3D electroanatomic voltage/local activation time map evaluations. Workflow, procedural data, and peri-procedural technical errors and complications are described.

**Results:**

Planned ablation strategy was achieved with FPFA-only in 48/51 (94%) of the cases. Ablation strategy was first-time pulmonary vein isolation (PVI) in 17/51 (36%), repeat ablation in 18/51 (38%), PVI + in 13/51 (28%), and cavotricuspid isthmus block (CTI)-only in 3/51 (6%). The mean procedure time was 104 ± 31 min (first-time PVI), 114 ± 26 min (repeat procedure), 152 ± 36 min (PVI +), and 62 ± 17 min (CTI). Mean UHDx mapping time to assess lesion formation and block after ablation was 7 ± 4 min with 5485 ± 4809 points. First pass acute (linear) isolation with bidirectional block for anatomical lesion sets was 120/124 (97%) for all PVs, 17/17 (100%) for (any) isthmus, and 14/17 (82%) for left atrium posterior wall (LAPW). We observed several time-consuming integration errors with the used ablation system (mean 3.4 ± 3.7 errors/procedure), one transient inferior ST elevation when ablating CTI resolved by intravenous nitroglycerine and one transient AV block requiring temporary pacing for > 24 h.

**Conclusions:**

FPFA was a highly versatile method to treat atrial arrhythmias with high first-pass efficiency. UHDx revealed acute homogenous low-voltage lesions in ablated areas. More data is needed to establish lesion durability and limitations of FPFA.

## Introduction

Recently, pulsed field ablation (PFA) has been introduced as a novel “single-shot” ablation method to achieve isolation of the pulmonary veins [1–5]. Potentially, this new method reduces the risk of collateral organ tissue damage when ablating in the heart, limiting feared complications such as damage to the oesophagus and phrenic nerves and pulmonary vein stenosis [6–9]. Prospective results and recurrence rates using three different multielectrode PFA catheters designed for “single-shot” pulmonary vein isolation (PVI) have now been reported [1, 4, 5, 10–13]. Although the 1-year recurrence rates seem comparable to thermal ablation methods, lesion durability, predictability, and safety profile of the systems make PFA very promising. However, so far, the possibility of creating designed ablation lesion sets targeting specific areas of interest, to treat critical isthmuses and have an integration with mapping systems, has been limited in the multielectrode systems. Now the first commercial system for focal PFA (FPFA) has been released consisting of a proprietary generator (CENTAURI, Galvanize EP) which can be coupled to specified ablation catheters and 3D mapping systems. CE-approval followed the data presented from the ECLIPSE AF trial (A. Anic et al. “Pulsed Electric Field Ablation for Pulmonary Vein Isolation (PVI): 90‐Day Remapping Results Using Three Compatible Focal Cardiac Ablation Catheters in the ECLIPSE AF Study”, presented at Heart Rhythm Society, 2022) following initial published pre-clinical data [14]. With this technology becoming readily available for ablation centres worldwide, there is a need to report independent, non-industry sponsored, experience with safety and procedural efficacy with FPFA. Furthermore, assessment of FPFA using detailed mapping systems is needed for optimal individually tailored treatment of all atrial arrhythmias and evaluation of lesion formation.

## Methods

### Patient selection for FPFA

The study was a prospective inclusion of an all-comer patient cohort for either first-time PVI, repeat procedure or treatment of other atrial arrhythmias. Inclusion was based in a single high-volume referral centre from October 27th, 2022, to March 22nd, 2023. Choice of FPFA PVI was based on the availability of general anaesthesia at our institution. Analysis of procedural and demographic anonymized data was approved by Herlev-Gentofte University Hospital Institutional Review Board (Case Number: 22035743).

### Procedural outline and setup

Standard computed tomography (CT) angiography of the left atrium (LA) and transoesophageal echocardiography with focus on interatrial septum and the LA appendage for thrombus assessment were performed 1 to 3 days in advance to the procedure. On the day of the procedure, all patients were fasting, on uninterrupted direct oral anticoagulant or vitamin K antagonists. Patients were intubated under general anaesthesia without use of paralytics. Five experienced electrophysiologists trained in multielectrode PFA, radiofrequency (RFA), and cryoballoon ablations performed the procedures. Three right femoral vein punctures, with or without ultrasound guidance by discretion of the operator, were obtained and a deflectable decapolar diagnostic catheter was placed in the coronary sinus (CS). For all the left atrial arrhythmias, a transseptal access was achieved by fluoroscopy and pressure-guided transseptal-puncture by use of SL1-sheath and BRK-1 needle as per standard. After access to the LA, systemic heparinization was done with target activated clotting time (ACT) 300–400 s. A NAVISTAR SmartTouch irrigated catheter (Biosense Webster, Irvine, CA, USA) was hereafter introduced to the LA through a steerable sheath. Depending on the planned procedure, a 3D ultrahigh-density (UHDx) bipolar voltage amplitude map and anatomical map were created with penta- or octa-spline multielectrode catheters (Biosense Webster) prior to final decision of an ablation strategy classified for the purpose of this study as either PVI, PVI + (with supplementary lesion sets), repeat procedure, or cavotricuspid-isthmus block (CTI-B)-only. Mapping was done during CS pacing and if patient was in AF from start — a DC conversion was done before mapping initiation. The CENTAURI generator (Galvanize EP) was used and connected as described [14] to the CARTO 3D mapping system (Biosense Webster). Ablation applications were ECG R-synchronized trains of pulsed field with a setting of 25 amperes for anterior wall/roof, CTI, and mitral isthmuses and 22 amperes for posterior wall. For 25 and 22 amperes, 10 and 7 pulse trains were delivered. Irrigation rate was 4 mL/h. In general, applications/tags of 6 mm were placed using the CLOSE protocol [15] but with 20–30% overlap based on the ECLIPSE AF data. For PVI, wide area circumferential ablation (WACA) was targeted. For repeat procedures, a qualitative assessment of the initial UHDx voltage map for determination on isolation, voltage, and scars was made along with formal testing of entry and exit blocks to the veins; thereafter, attempted induction of trigger activity or atrial tachyarrythmias with isoprenaline infusion to a heart rate > 100 bpm and atrial burst pacing was initiated. Hereafter, ad hoc ablation based on the above findings was performed for the repeat procedures. In case of isthmus ablation, an administration of intravenous nitroglycerine 0.2 mg over 1 min was given prior to ablation with an additional dose of 0.2 mg if possible after (any) hypotension had been resolved by concomitant vasopressor and/or saline infusion. Atrial tachycardias were mapped accordingly using local activation time maps and coherence conduction vectors to establish critical isthmuses and circuits with supplementary entrainment mapping if needed as per clinical standard and described [10, 16–18]. Following ablations, one or more confirmatory detailed UHDx bipolar voltage amplitude 3D maps of the ablation line(s) in LA or right atrium were performed with the multielectrode catheter using proximal CS pacing. Supplementary paced local activation time evaluation over ablation lines could be applied in cases of isthmus block if deemed of relevance. The colour display range of the bipolar voltage map was set to 0.2 to 0.5 mV to visualize gaps, zones of healthy tissue, and low-voltage areas. After the map, a qualitative evaluation of the performed lesion set/ablations line(s) was done along with adenosine testing for dormant conduction. Follow-up of complications up to 14 days after the procedure was available through chart review.

### Statistical analysis

For continuous variables, the mean ± SD or median ± ÍQR were used as appropriate. For categorical values, the number and percentages were used.

## Results

A total of 51 patients with atrial fibrillation (AF) or atrial tachycardia (AT) (paroxysmal AF = 29, persistent AF = 19) with overlapping diagnoses of AT, atypical flutter, and typical flutter were included to receive ablation treatment with FPFA. It was first-time ablation procedure for 33/51 (65%) and a repeat procedure for 18/51 (35%). Baseline patient characteristics are presented in Table 1. In summary, patients were 65 ± 12 years old, 67% were male, average BMI was 27.7 ± 4.3 kg/m^2^, 49% had hypertension, and 16% had heart failure. The mean left ventricular ejection fraction was 55 ± 9% and 57% had moderately or severely dilated LA. Concomitant pharmacotherapy at time of procedure included beta-blockers (82%), flecainide (14%), and amiodarone (25%).

**Table 1:** Baseline characteristics

Baseline characteristics	*n* = 51
Age, years	65 ± 12
Male sex, *n* (%)	34 (67)
BMI, kg/m^2^	27.7 ± 4.3
Type of AF	
Paroxysmal, *n* (%)	29 (57)
Persistent, *n* (%)	19 (37)
Atrial tachycardia or atrial flutter	18 (35)
EHRA class	2.5 ± 0.6
NYHA class	1.4 ± 0.5
Coronary artery disease, *n* (%)	2 (4)
Diabetes, *n* (%)	2 (4)
Hypertension, *n* (%)	25 (49)
Heart failure, *n* (%)	8 (16)
Previous stroke, *n* (%)	5 (10)
CHADSVASc score	1.9 ± 1.3
Left ventricular ejection fraction, %	55 ± 9
Left atrium moderately or severely dilated, *n* (%)	29 (57)
History or current use of	
Beta-blockers, *n* (%)	42 (82)
Amiodarone, *n* (%)	13 (25)
Flecainide, *n* (%)	7 (14)
Class IV calcium channel blockers, *n* (%)	1 (2)
Direct oral anticoagulant, *n* (%)	46 (90)
Implantable device including loop recorders	2 (4)

Values are in mean ± SD. *AF*, atrial fibrillation; *BMI*, body mass index; *EHRA*, European Heart Rhythm Association; *NYHA*, New York Heart Association

### Procedural results

The 51 procedures were performed by five electrophysiologists experienced with multielectrode PFA, cryoballoon, and RFA, distributed case numbers 13, 12, 11, 10, and 5. All patients were under general anaesthesia. Overall mean procedure time was 118 ± 37 min (median 112 min) and mean fluoroscopy time was 6 ± 3 min (Table 2). The planned ablation strategy was achieved with FPFA-only in 48/51 (94%) of the cases. In two of the three cases where FPFA was supplemented by RFA was because of safety-concerns of potential risk of coronary spasm when ablating a CTI line in a patient with severe coronary artery disease and in one case of an anterior mitral isthmus line. The third use of hybrid FPFA and RFA was because of technical error and shut-down of the FPFA system. Ablation strategy was first-time PVI in 17/51 (36%), repeat ablation in 18/51 (38%), PVI with additional lesion set (PVI +) in 13/51 (28%), and CTI-B only in 3/51 (6%). A total of 100% of the cases were evaluated with UHDx maps. Average post-ablation mapping time was 7 ± 4 min and mean number of mapping points was 5485 ± 4809. The overall mean number of FPFA applications (tags) was 53 ± 27 and mean time from first application to last FPFA application was 47 ± 29 min as a surrogate marker of ablation time.

**Table 2:** Overall procedural data

Procedural characteristics	Total
General anaesthesia/intubation	51/51 (100%)
Procedure time (skin to skin), mean ± SD, min	118 ± 37
Procedure time (skin to skin), median (IQR), min	112 (88–135)
Fluoroscopy, mean ± SD, min	6 ± 3
Fluoroscopy, median (IQR), min	5 (4–8)
Dose area product, mean ± SD, cGy × cm^2^	9 ± 10
First-time PVI	17/51 (36%)
Repeat ablation	18/51 (38%)
First-time PVI with additional lesion set – PVI ( +)	13/51 (28%)
Cavotricuspid isthmus block – only	3/51 (6%)
Isolation achieved with FPFA*-only, patients (%)	48/51 (94%)
FPFA applications (tags), mean ± SD, min	53 ± 27
Ultrahigh-density 3D bipolar voltage maps	51/51
Post ablation evaluation mapping time, mean ± SD, min	7 ± 4
Mapping points, mean ± SD, min	5485 ± 4809

^*^Two patients were selected for hybrid FPFA and radiofrequency (RF) approach because of coronary spasm concerns. In one patient, a switch to RF was necessary due to technical errors of the PFA system

*DAP*, dose area product; *IQR*, interquartile range; *SD*, standard deviation; *FPFA*, focal pulsed field ablation; *PVI*, pulmonary vein isolation

### Pulmonary vein isolation

**Fig. 1:**
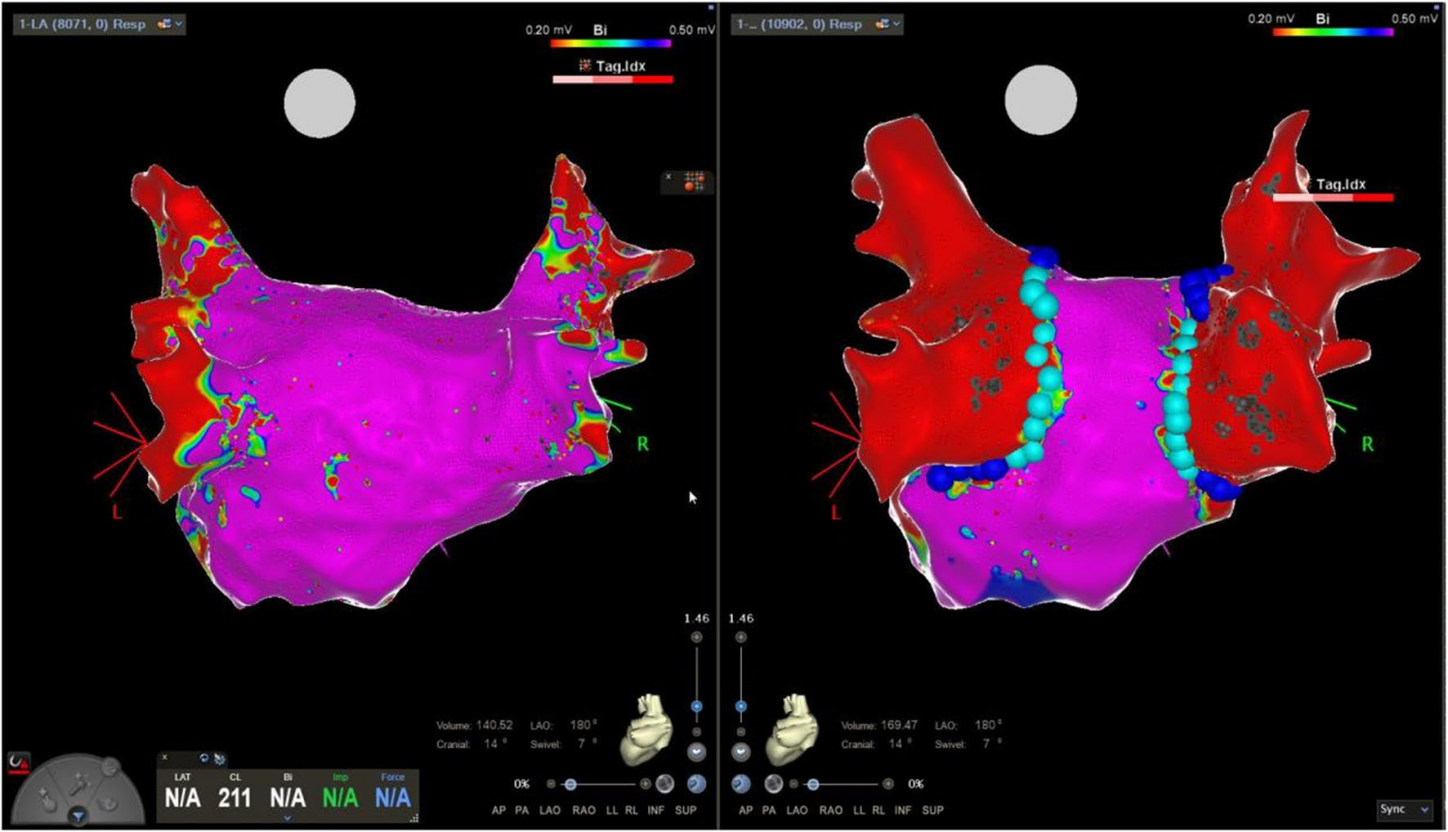
First-time pulmonary vein isolation. Pre-ablation and post-ablation voltage maps. Left panel shows pre-ablation bipolar voltage map of the posterior aspect of the left atrium. The right panel shows same perspective post-ablation with focal pulsed field ablation applications/tags. Dark blue tags 25A energy delivery setting and cyan colour 22A energy delivery setting. No sign of fractionation or low voltage in the proximity to the sharply demarcated ablation lines. Colour coding settings for bipolar voltage from 0.2 mV (red) to 0.5 mV (magenta)

**Fig. 2:**
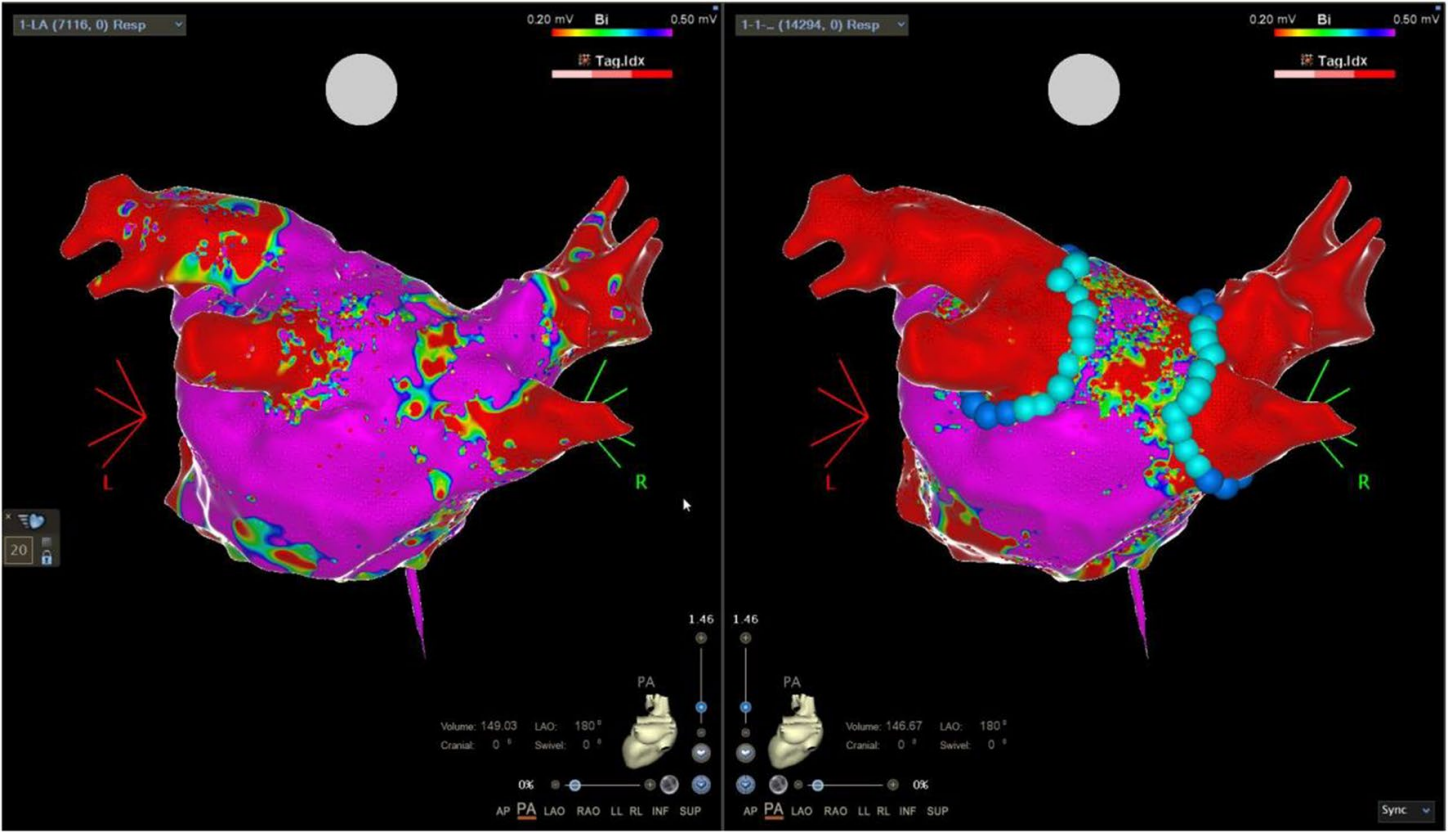
First-time pulmonary vein isolation. Pre-ablation and post-ablation voltage maps. Left panel shows pre-ablation bipolar voltage map of the posterior aspect of the left atrium. The right panel shows same perspective post-ablation with focal pulsed field ablation applications/tags. Dark blue tags 25A energy delivery setting and cyan colour 22A energy delivery setting. Evidence of increased low voltage in the proximity to the ablation lines covering large parts of the posterior wall. Colour coding settings for bipolar voltage from 0.2 mV (red) to 0.5 mV (magenta)

A total of 30 first-time PVIs were ablated, of which 17 (57%) were PVI-only and 13 (43%) were PVI with additional lesion set (PVI +). Complete PVI was achieved with FPFA-only in 29/30 (97%) patients for the reasons described above. First-pass isolation was achieved in 58/60 (97%) of the left-sided veins and in 64/64 (100%) of the right-sided veins (total 122 of 124). The mean number of PVI applications/tags was 65 ± 8 WACA lesions with an average “treatment” time of 46 ± 12 min from first application/tag to last application/tag for PVI-only. Mean skin to skin procedure time was 106 ± 31 min for PVI-only. Post-PVI UHDx mapping (mean 7 ± 4 min) showed WACA lines with sharp demarcation of lesion border zones with no apparent areas of complex electrogram-fractionation in most of the cases (26/30) (Fig. 1). We, however, also observed 4/30 (13%) cases with increased areas of low voltage on the left atrial posterior wall (LAPW) of unknown significance in the border zones of the application/tags (Fig. 2). No fractionation was observed in these 4 cases. Acute reconduction was found in 1/124 veins (1%) located anteriorly-superiorly in the right superior PV during post-PVI mapping. Supplementary adenosine testing for dormant conduction after mapping was negative in 64/64 (100%) of the tested veins (Table 3).

**Table 3:** Procedural parameters by type of procedure

	PVI-only	Repeat procedure	PVI ( +) additional lesions	Any LAPWi (first and repeat)	Any isthmus (first and repeat)	Any PVI (not repeat)
Number of patients *Overlap between groups	17	18	13	17	17	30
Procedure time (skin to skin), mean ± SD, min	106 ± 31	114 ± 26	152 ± 36	125 ± 33	118 ± 37	125 ± 40
Fluoroscopy, mean ± SD, min	6 ± 3	7 ± 3	6 ± 3	7 ± 3	6 ± 4	6 ± 4
Dose area product, mean ± SD, cGy × cm^2^	6 ± 5	13 ± 14	9 ± 9	11 ± 14	11 ± 14	8 ± 7
First-time PVI	17	0	13	5	8	30
Repeat ablation, #	0	18	0	12	6	0
FPFA applications (tags), mean ± SD, min	65 ± 7	32 ± 16	83 ± 16	24 ± 12	10 ± 4	65 ± 8
Ultrahigh-density 3D mapping points, mean ± SD	4131 ± 2922	8104 ± 7542	5508 ± 3900	6994 ± 7487	6071 ± 3473	4683 ± 3252
Post ablation evaluation mapping time, mean ± SD, min	7 ± 5	9 ± 3	7 ± 3	8 ± 3	8 ± 3	7 ± 4
First pass isolation or linear anatomical block, anatomical sites, #	69/70 (99%)	23/25 (92%)	59/60 (98%)	14/17 (82%)	17/17 (100%)	122/124 (98%)
Adenosine positive for dormant conduction, #	0/40 (0%)	0/8 (0%)	0/5 (0%)	0/9 (0%)	0/6 (0%)	0/64 (0%)

*DAP*, dose area product; *SD*, standard deviation; *PVI*, pulmonary vein isolation; *PVI (* +*)*, pulmonary vein isolation with additional lesion sets; *LAPW*, left atrium posterior wall isolation; *FPFA*, focal pulsed field ablation

### Left atrium posterior wall isolation

**Fig. 3:**
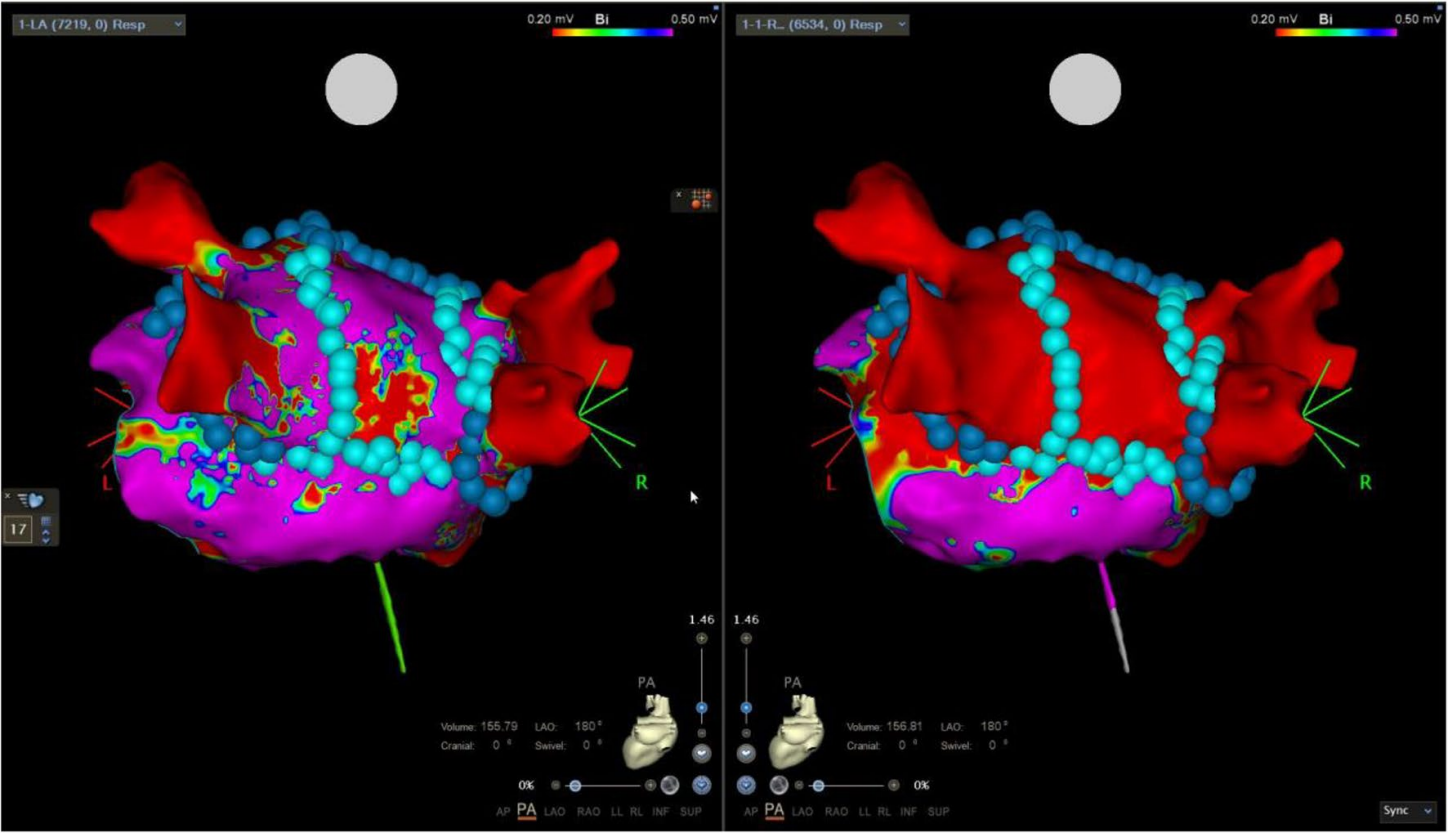
First-time pulmonary vein isolation with additional left atrium posterior wall isolation. Pre-ablation and post-ablation voltage maps. Left panel shows pre-ablation bipolar voltage map of the posterior aspect of the left atrium including the subsequent ablation tags. The low bipolar voltage indicated a large scar on the posterior wall. The right panel shows same perspective post-ablation now with isolated pulmonary veins and posterior wall. Dark blue tags 25A energy delivery setting and cyan colour 22A energy delivery setting. Colour coding settings for bipolar voltage from 0.2 mV (red) to 0.5 mV (magenta)

**Fig. 4:**
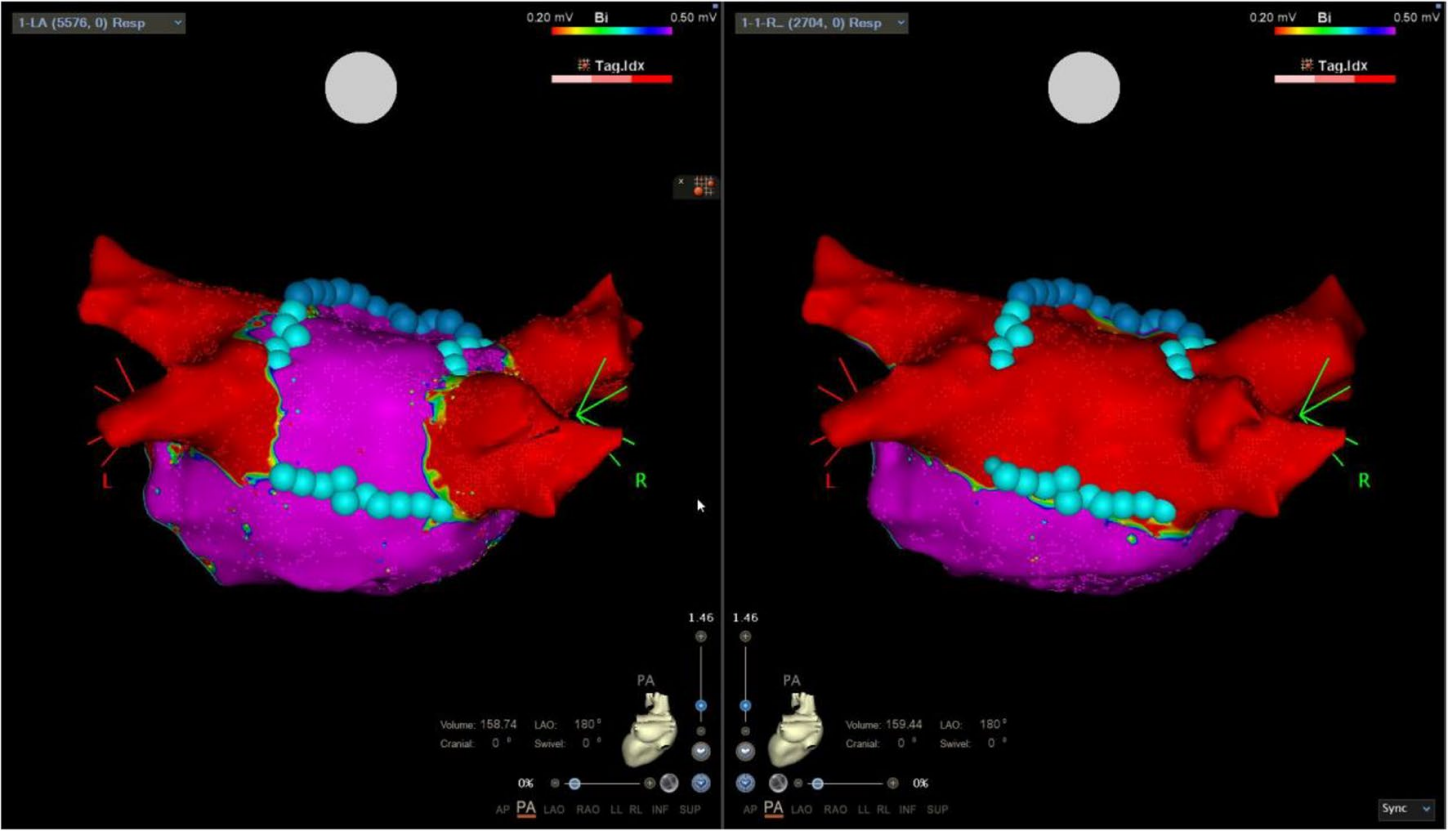
Left atrium posterior wall isolation in a repeat procedure with durably isolated pulmonary veins. Pre-ablation and post-ablation voltage maps. Left panel shows pre-ablation bipolar voltage map of the posterior aspect of the left atrium with confirmation of isolated pulmonary veins. The right panel shows same perspective post-ablation with isolation of the posterior wall and additional applications in both carina regions. Dark blue tags 25A energy delivery setting and cyan colour 22A energy delivery setting. Colour coding settings for bipolar voltage from 0.2 mV (red) to 0.5 mV (magenta)

A total of 17 left atrium posterior wall isolations (LAPWi) were completed, of which 12/17 (71%) were as repeat procedures and 5/17 (29%) were part of a PVI ( +) strategy. Using roof zenith and inferior nadir linear ablation lines (Figs. 3 and 4) resulted in first-pass linear isolation in 14/17 (82%), while additional ablation of epicardially connected fibres was necessary in the LAPW centre zone in 3/17 (18%). Mean roof-line and inferior line number of applications/tags were 18 ± 11 and 12 ± 1, respectively, while 4, 4, and 8 additional applications were used in the centre zone for isolation in the three cases of no linear first-pass isolation. No acute reconnection or dormant conduction was observed in any of the 17 LAPWi and mapping (mean points 6993 ± 7487) showed sharp demarcation of lesion border zones with no apparent areas of complex electrogram-fractionation.

### Isthmus lines

**Fig. 5:**
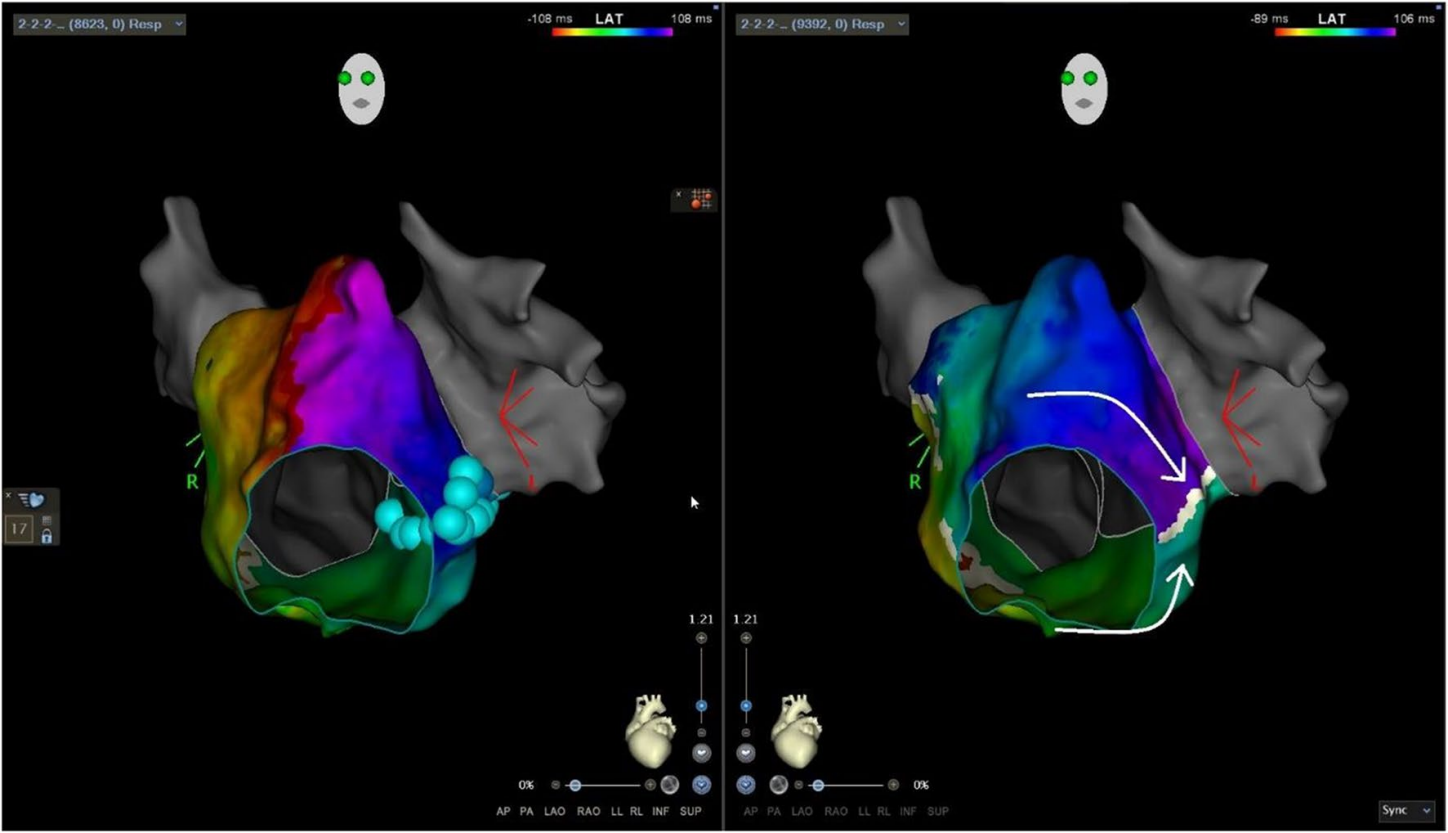
Lateral mitral-isthmus ablation line. Pre-ablation and post-ablation maps. Left panel shows pre-ablation local activation time (LAT) map showing a counter-clockwise perimitral flutter including application/tags where for this case cyan colour indicated 25A energy delivery setting. Right panel shows CS paced map with line of block at the ablated site

In context of PVI( +), as part of repeat procedure or as a stand-alone procedure, a total of 17 isthmus lines were created with FPFA due to macro-reentry tachycardias, 12 CTI-B, and four mitral-isthmus blocks (MIB) (three anterior lines and one lateral) (Fig. 5). In all cases of ongoing macro-reentry tachycardias, we experienced acute termination following FPFA of the intended line. The mean number of FPA applications/tags was 9 ± 3 and 13 ± 4 for CTIB and MIB, respectively. After anatomical linear line, a formal block was confirmed by pacing manoeuvers in 17/17 (100%) and CS paced UHDx maps (mean mapping points 6071 ± 4373, mean mapping time 8 ± 3 min) showed sharp demarcation of lesion border zones and propagation block on the line. In one repeat procedure, a focal atrial tachycardia was mapped according to earliest activation to the mid portion of the terminal crest in the right atrium. Ongoing tachycardia was terminated upon first application of FPFA to the area, and was hereafter non-inducible.

### Complications

The acute complications are presented in Table 4. We observed two complications related to the procedures. One case of prolonged complete atrioventricular (AV) block in a patient with first-degree AV block present at the beginning of the procedure, necessitating a temporary pacemaker for 24 h, but hereafter resolution of the block. During the PVI and ablation of the right sided veins — first progression to a left bundle branch block became apparent. Shortly after procedure termination, a further progression of the conduction delay led to second-degree AV block and then complete AV block. One case of ST-elevations in the inferior leads was also observed despite an initial dose of 0.2 mg of nitroglycerine had been given prophylactic before initiation of a CTI-B. ST-elevations occurred approximately 5 min after the administration during FPFA applications. An additional 0.2-mg nitroglycerine administration resolved the ST-elevations and no further problems were seen.

**Table 4:** Procedural complications within 14 days

Adverse event	Major complications	Minor complications
Tamponade	0	
Stroke	0	
Phrenic nerve palsy	0	
Atrioventricular block > 24 h requiring temporary pacing	1	
Haemoptysis		0
Pericarditis		0
Pneumonia		0
Vascular complications		0
ST elevations in inferior leads, resolved by nitroglycerine		1
Total	1 (2%)	1 (2%)

FPFA to the right sided veins resulted in phrenic nerve capture, but no phrenic nerve palsies were observed. In one case of roof-line FPFA applications, singular premature ventricular complexes were seen after every application but resolved hereafter. We observed no ventricular arrhythmias, no strokes, tamponades or evidence of oesophageal injury, and no deaths related to the procedures. There were no vascular complications leading to hospitalization or interventions; however, 1 (2%) vascular hematoma was observed and handled conservatively.

### Integration errors

Over the course of the 51 ablation procedures, several errors and issues were noted mainly due to integration errors with the mapping system (Table 5). Some errors were more prevalent and time-consuming than others. After every FPFA application, the system must restore connection resulting in disappearance of the catheter and catheter parameters in the 3D mapping system and a re-visualization of catheter parameters is initiated (error 2604). In a representative sample of 10 FPFA applications, we estimated the time from last FPFA application to the catheter was visible again with available parameters and ready to move to 14 ± 8 s. With an average number of tags/applications for standard PVI of 65, this is potentially more than 15 min of catheter recovery time — which is in line with our experiences. Another compatibility issue was a patch error unit (error 1003), resolved by physical disconnection and reconnection of the patch unit cable. This error was found to be more prevalent the more applications were applied (*R*^2^ = 0.48). The average number of patch unit error per case was 3.4 ± 3.7. In two cases, asynchronous FFPA applications were necessary due to ECG synchronization error. In additional five cases, various heterogeneously distributed errors were observed without any apparent (i.e. errors 1011, 1012) systematic cause resolved typically by shut-down and re-booting of the patient interface unit (PIU). In one case, as previously mentioned, an integration error of undetermined cause resulted in shut-down of the PFA system-mapping system compatibility and a change to RFA-only was necessary.

**Table 5:** Integration errors and possible solutions

Error	Frequency	Solution
Catheter visualization (error 2604)	After every FPFA application	Waiting 14 ± 8 s
Patch unit error (error 103)	3.4 ± 3.7 times per case	Manually disconnect and reconnect patch unit cable
ECG synchronization error	Infrequent	Adjust CENTAURI generator to asynchronous FPFA delivery
Errors 1011, 1012 etc	Infrequent	Switch the Patient Interface Unit (PIU) off and on

## Discussion

This report outlines the initial experiences, feasibility, acute procedural success, safety, and UHDx mapping evaluations of a variety of ablation procedures all treated with FPFA. The main findings were a high rate of acute procedural success with very high first-pass efficiency for PVI, LAPWi, and anatomical lines with a low rate of adverse events. The integration with a mapping system allowed individually tailored FPFA treatment resulting in versatile, flexible, and high acute treatment success of all types of observed atrial arrhythmias. This is the first study of FPFA to evaluate acute procedural results for a broad spectrum of consecutive atrial arrhythmias.

### Pulmonary vein isolation

Using UHDx mapping, we found that tissue voltage was reduced to minimum values in the ablated areas resulting in WACA for PVI and conduction blocks for linear ablation lines. Whether this results in durable lesions or is part of a stunning phenomenon remains to be explored. It is important to keep in mind that acute isolation is not a surrogate for durable isolation or freedom from AF for that matter. The preliminary data from the ECLIPSE-AF trial presented at HRS 2022 was however promising, and suggested a 90-day PVI durability of 92% (*n* = 63) (Anic A. et al. Pulsed Electric Field Ablation for Pulmonary Vein Isolation (PVI): 90‐Day Remapping Results Using Three Compatible Focal Cardiac Ablation Catheters in the ECLIPSE AF Study; abstract presented at HRS 2022). Importantly, in the present non-industry sponsored all-comer atrial arrhythmia population, we were able to demonstrate similar first-pass acute success and efficiency for PVI as presented in ECLIPSE-AF. In a recent publication from our group, we saw that high-density 3D mapping evaluation after PVI by multielectrode PFA resulted in acute PVI but insufficient WACA lesion sets and particularly right sided gaps resulting in additional applications of PFA in up to 20% [16], which calls for caution on the termed success of acute PFA isolation. Bohnen et al. found insufficient lesion formation after PFA PVI more often located in the anterior antral parts of the left sided veins [19], while Gunawardene et al. found early PV-reconnection in 5/80 (6.3%) all located in the anterior–superior PV ostia on both left and right sides [20]. Thus, so far, there is inconclusive evidence of the acute and chronic lesion formation as well as the antral WACA coverage after multielectrode PFA in all-comer AF populations. Furthermore, in the only published series of recurrent AT in repeat procedures after PVI with multielectrode PFA performed under fluoroscopy, without mapping guidance, there seems to be a predominance of roof-dependent ATs fuelled by narrow isthmus formations of the posterior wall [21]. The PV reconnection rate was 9% in that study (*n* = 25). In this series of PVI — we only observed acute reconduction in one of 124 PVs despite using both the observation time of additional lesion set ablations (for PVI ( +)), detailed mapping and supplementary adenosine testing for dormant conduction.

### Macro-reentry tachcardias

Integration of FPFA with UHDx mapping systems allows for detailed identification of scar tissue and identification of AT mechanisms and HD mapping has been shown to improve detection of areas critical to maintain AT circuits [22]. We saw acute FPFA termination of focal atrial and macro-reentry tachycardias in all cases and achieved acute formally confirmed block in all isthmus lines. In addition, we saw no reconnections during the mapping time for the treated anatomical isthmuses. Again, whether this is due to a temporary stunning effect or a result of potential durable lines remains to be explored. Previous reports showed only 58% complete block of an anterior mitral-isthmus line when treated with RFA [23], while more recent data from the temperature-controlled lattice-tip focal RFA catheter, however, showed very high mitral-isthmus line durability of 91% (*n* = 11) and 100% for CTI lines (*n* = 25). Similarly, present FPFA results are also encouraging of achieving successful anatomical linear blocks. In the PersAFone study [24], acute CTI bidirectional block was achieved in 13 of 13 (100%) patients using the same large focal lattice-type catheter as above but utilizing PFA and was in that study delivered at a median of 6 sites (IQR: 5 to 7 sites) per CTI with an average of 9 min (IQR: 6 to 12 min) between the first and last PFA deliveries. Follow-up durability mapping of CTI from PersAFone at a median of 87 days (IQR: 76 to 90 days) after index procedure showed 25% durability for the first four patients who were exposed to a lower initial PFA dose (called Focal-1 of 1.6 kV) compared to 100% durability in eight out of eight patients with Focal-2 of 1.8 kV of PFA dosing. To our knowledge, there has not been published any repeat mapping procedures after PFA of the mitral isthmus. As mentioned, we also successfully ablated one focal atrial tachycardia from the terminal crest in the right atrium as part of PVI ( +). Recently, a case report highlighted the possibilities of successful FPFA of an AT in close proximity to the phrenic nerve [25].

### Left atrium posterior wall

Although multielectrode PFA has been shown to be useful and feasible for successful ablation of the LAPW with or without supplementary mapping [10, 16, 24], feedback on contact and force values may be important for optimal and durable lesion formation of PFA [26]. These parameters are not yet available in clinical practice. In 21/21 (100%) invasive remapping procedures performed after 82 (IQR 76–90) days, the LAPW was found durably isolated in the PersAFone study [24]. Compared to our recent published experiences with RFA-based LAPWi, where as many as 33% required ablations in the centre PW for isolation and a rather low 46% LAPWi durability rate at 6 months at the cost of asymptomatic, yet worrisome, oesophageal lesions [27, 28], we find LAPWi with FPFA highly effective. Oesophageal safety for FPFA has not been fully established but so far preclinical and clinical data from multielectrode PFA seem convincing and reassuring [3, 7, 24]. Since no trial has yet established LAPWi as a superior ablation strategy on top of standard PVI for persistent AF [29], speculations on causal issues such as the low durability of the LAPWi arise, which may now be potentially partly solved by PFA. This remains to be proven in a randomized trial. Furthermore, the utility of LAPWi as step two for repeat procedures with durable PVI remains to be proven in a randomized trial, most preferably with a method that has a proven durable lesion set. Although we cannot correlate that combination of detailed mapping and FPFA leads to improved outcomes, we found that supplementary LAPWi was feasible with high acute isolation rate for persistent and long-standing persistent AF patients but also as a strategy for supplementary ablation treatment in repeat procedures with LAPW scar fractionation and/or durably isolated veins from initial PVI.

### Feasibility/safety

We found a low rate of acute complications. Being well aware of the issues of reported coronary spasms when performing PFA close to the coronary arteries [11], administration of nitroglycerine was done upfront. In one case, there were still ST-elevations which resolved by further treatment with nitroglycerine and this issue remains to be further evaluated in larger series. The apparent stunning of the conduction system and temporary AV block we experienced when ablating closer to the septum is also of concern and needs further exploration. The observed complication occurred in a patient with 1st-degree AV block present prior to the procedure. Whether there is increased risk of AV block in patients predisposed to intraatrial, septal, and AV node conduction delay remains to be evaluated in larger series. The right atrium was not mapped in the patient but no apparent septal fibrotic tissue was present in the LA map and the WACA spatial line around the right septal veins was “standard”. Mapping and integration errors reflect a 1st-generation system as can be expected. Currently, the procedure time and technical skills required for point-by-point ablation still pose a limitation of FPFA for standard PVI in comparison to single-shot PFA techniques. In contrast, the combination of UHDx and detailed AT circuit mapping with FPFA treatment tailored to area-specific targets with a high first-pass and acute termination effect seems promising for both repeat procedures and for first-time AT ablation. Furthermore, this approach can be performed with low fluoroscopy doses but point-by-point ablations may require a prolonged learning curve and longer procedure times than single-shot. Recently, the SmartfiRE trial was initiated aiming to include 135 participants in a prospective evaluation of another FPFA system (clinicaltrials.gov NCT 05752487) while the publication and recurrence rates of the ECLIPSE-AF trial are eagerly awaited.

## Limitations

This is a real-life application of a newly introduced and commercially available ablation technique. The small sample size limits the conclusions on safety and it is too early to report credible recurrence rates. Being a single-centre study, this limits the applicability to other centres. However, the present study reports the safety, feasibility, and acute efficacy of the present PFA system and cannot be extrapolated to other PFA systems.

## Conclusions

FPFA was a highly versatile method to treat all encountered atrial arrhythmias with high first-pass efficiency for PVI as well as successful creation of LAPWi and atrial isthmus blocks and acute termination of ATs. UHDx was used effectively to map ATs and revealed acute homogenous low-voltage lesions in ablated target areas. More data is needed to establish lesion durability and limitations of FPFA.
